# Pharmacological characterisation of CR6086, a potent prostaglandin E_2_ receptor 4 antagonist, as a new potential disease-modifying anti-rheumatic drug

**DOI:** 10.1186/s13075-018-1537-8

**Published:** 2018-03-01

**Authors:** Gianfranco Caselli, Albino Bonazzi, Marco Lanza, Flora Ferrari, Daniele Maggioni, Cristian Ferioli, Roberto Giambelli, Eleonora Comi, Silvia Zerbi, Marco Perrella, Ornella Letari, Elena Di Luccio, Milena Colovic, Stefano Persiani, Tiziano Zanelli, Laura Mennuni, Tiziana Piepoli, Lucio Claudio Rovati

**Affiliations:** Rottapharm Biotech, Via Valosa di Sopra 9, I-20900 Monza, MB Italy

**Keywords:** Arthritis, EP4 receptor antagonist, CR6086, Immunomodulatory potential, Collagen-induced arthritis

## Abstract

**Background:**

Prostaglandin E_2_ (PGE_2_) acts via its EP4 receptor as a cytokine amplifier (e.g., interleukin [IL]-6) and induces the differentiation and expansion of inflammatory T-helper (Th) lymphocytes. These mechanisms play a key role in the onset and progression of rheumatoid arthritis (RA). We present the pharmacological characterisation of CR6086, a novel EP4 receptor antagonist, and provide evidence for its potential as a disease-modifying anti-rheumatic drug (DMARD).

**Methods:**

CR6086 affinity and pharmacodynamics were studied in EP4-expressing HEK293 cells by radioligand binding and cyclic adenosine monophosphate (cAMP) production, respectively. In immune cells, IL-6 and vascular endothelial growth factor (VEGF) expression were analysed by RT-PCR, and IL-23 and IL-17 release were measured by enzyme-linked immunosorbent assay (ELISA). In collagen-induced arthritis (CIA) models, rats or mice were immunised with bovine collagen type II. Drugs were administered orally (etanercept and methotrexate intraperitoneally) starting at disease onset. Arthritis progression was evaluated by oedema, clinical score and histopathology. Anti-collagen II immunoglobulin G antibodies were measured by ELISA.

**Results:**

CR6086 showed selectivity and high affinity for the human EP4 receptor (*K*_i_ = 16.6 nM) and functioned as a pure antagonist (half-maximal inhibitory concentration, 22 nM) on PGE_2_-stimulated cAMP production. In models of human immune cells in culture, CR6086 reduced key cytokine players of RA (IL-6 and VEGF expression in macrophages, IL-23 release from dendritic cells, IL-17 release from Th17 cells). In the CIA model of RA in rats and mice, CR6086 significantly improved all features of arthritis: severity, histology, inflammation and pain. In rats, CR6086 was better than the selective cyclooxygenase-2 inhibitor rofecoxib and at least as effective as the Janus kinase inhibitor tofacitinib. In mice, CR6086 and the biologic DMARD etanercept were highly effective, whereas the non-steroidal anti-inflammatory drug naproxen was ineffective. Importantly, in a study of CR6086/methotrexate, combined treatment greatly improved the effect of a fully immunosuppressive dose of methotrexate.

**Conclusions:**

CR6086 is a novel, potent EP4 antagonist showing favourable immunomodulatory properties, striking DMARD effects in rodents, and anti-inflammatory activity targeted to immune-mediated inflammatory diseases and distinct from the general effects of cyclooxygenase inhibitors. These results support the clinical development of CR6086, both as a stand-alone DMARD and as a combination therapy with methotrexate. The proof-of-concept trial in patients with RA is ongoing.

**Electronic supplementary material:**

The online version of this article (10.1186/s13075-018-1537-8) contains supplementary material, which is available to authorized users.

## Background

Rheumatoid arthritis is an immune-mediated, systemic inflammatory disease that affects mainly synovial joints, with intra-articular inflammation, synovial hyperplasia and progressive degradation of cartilage and bone. Its prevalence is around 1% of the population, and the disease is more frequent (and perhaps worse) in women than in men. There have been clear advances in the pharmacological management of rheumatoid arthritis over the last decade, but many patients still do not tolerate or do not respond well to the available therapies. A vast pipeline of products under development substantiates that there is still a considerable unmet medical need in rheumatoid arthritis [1–4].

The current approach consists of tight control of disease activity through prompt institution of a disease-modifying anti-rheumatic drug (DMARD) therapy aimed at blocking disease progression and joint damage. Both the American College of Rheumatology and the European League Against Rheumatism recommend that treatment with DMARDs should start as soon as a diagnosis has been made [5, 6]. Traditional non-steroidal anti-inflammatory drugs (NSAIDs) and corticosteroids are reserved for symptomatic relief and bridging therapies [7].

Rheumatoid arthritis is characterised by activation of different immune cell types with distinct cytokine profiles and effector functions. In addition, joint cells, such as osteoblasts, fibroblast-like synoviocytes and chondrocytes, play a key role in the inflammatory/erosive pattern that characterises the disease beginning in its early stages. Among the multiple targets involved in the pathogenesis of rheumatoid arthritis, the prostaglandin E_2_ receptor 4 (EP4) subtype receptor of prostaglandin E_2_ (PGE_2_) is one of the most promising because, unlike common NSAIDs that inhibit the synthesis of prostaglandins, selective EP4 antagonists have the potential to combine immunomodulatory and direct anti-inflammatory properties [2, 8–11].

Growing evidence in the literature supports this assumption. EP4 is expressed by macrophages, and its activation works as a cytokine amplifier (e.g., interleukin [IL]-6 expression), a response inhibited by EP4 antagonists [12, 13]. EP4 signalling promotes IL-23 synthesis by dendritic cells and their chemotaxis from peripheral tissues to draining lymph nodes, a crucial step for the priming of T cells [14–17]. Importantly, PGE_2_-EP4 interaction facilitates T-helper type 1 (Th1) lymphocyte differentiation and Th17 expansion [8, 14, 18, 19]. The receptor is generally present even in joint cells, including osteoblasts [20], osteoclasts [12], chondrocytes and fibroblast-like synoviocytes [13]. EP4 signalling is a major player in the bone resorption associated with rheumatoid arthritis because it enhances osteoclast formation and activation [21].

In line with these findings, PGE_2_ exacerbates collagen-induced arthritis (CIA) in mice [22]. Notably, in a model of collagen antibody-induced arthritis, EP4-knockout mice were resistant to the development of experimental arthritis. Clinical, histopathological and cellular markers of the disease were significantly less severe in EP4 receptor-deficient mice than in wild-type controls or in EP1–EP3-knockout mice [18].

We present the pharmacological characterisation of CR6086 (chemical structure provided in Additional file 1), a potent and selective EP4 receptor antagonist, and provide evidence for its potential as a DMARD. CR6086 showed favourable immunomodulatory properties, striking disease-modifying effects in rodent models of rheumatoid arthritis (with promising results against immune-mediated disruption of physiologic bone remodelling), and anti-inflammatory activity targeted to immune-mediated inflammatory diseases and distinct from the general effects of cyclooxygenase (COX) inhibitors.

## Methods

### Expression of EP4 receptors in human embryonic kidney cell line

The complementary DNA (cDNA) clone of the human EP4 receptor (hEP4R; NM_000958.2) was obtained from Thermo Fisher Scientific (Ultimate ORF Clone Collection, clone ID IOH46525; Waltham, MA, USA). The coding sequence was subcloned in expression vector pcDNATM6.2/V5-DEST by using Invitrogen Gateway technology (Thermo Fisher Scientific). Human embryonic kidney cells 293 (HEK293) were obtained from the American Type Culture Collection (ATCC; Manassas, VA, USA) and transfected with expression vector for hEP4R according to the method described in the FuGENE 6 Transfection Reagent technical manual (Promega, Madison, WI, USA). The clones overexpressing the protein were selected for Blasticidin S HCl (Thermo Fisher Scientific) resistance. The selected clone was grown in DMEM containing 10% FBS and 10 μg/ml Blasticidin S HCl (selection medium) at 37 °C in a humidified atmosphere of 5% CO_2_ in air.

For rodent EP4 receptor cloning, the cDNA encoding the full-length rat (NM_032076) or mouse (NM_001136079) EP4 receptors cloned in pCMV6 entry was provided by OriGene Inc. (TrueORF Gold, catalogue numbers RN206839 and MC216813; OriGene, Rockville, MD, USA). Expression of rat and mouse receptors was obtained by transfecting HEK293 cells with the respective vector using Lipofectamine 3000 reagent (Thermo Fisher Scientific) according to the manufacturer’s instructions.

### EP4 binding assay

#### Cell membrane preparations

Membranes expressing the human EP4 receptor were prepared from stably transfected HEK293 cell line. Cells were grown adherent in DMEM with GlutaMAX-I (Thermo Fisher Scientific) containing 10% FBS and Blasticidin 10 μg/ml at 37 °C in a humidified atmosphere with 5% CO_2_. Membranes expressing the mouse or rat EP4 receptor were prepared from transiently transfected HEK293 cells. Cells were grown adherent in DMEM with GlutaMAX-I containing 10% FBS at 37 °C in a humidified atmosphere with 5% CO_2_. For the preparation of all cell membranes, culture medium was aspirated from 150-cm^2^ flasks into which cells were seeded. Cell monolayers were then washed with 10 ml of hypotonic lysis buffer (Tris 5 mM + ethylenediaminetetraacetic acid [EDTA] 5 mM, pH 7.4), detached, and lysed with 10 ml + 10 ml of lysis buffer by mechanical scraping. Lysates were vortexed for 30 seconds and centrifuged at 40,000 × *g* for 22 minutes at 4 °C. Pellets were stored at −80 °C until use. Protein content of the cell membrane suspension was determined using bovine serum albumin (BSA) as a standard.

#### Radioligand binding assays

Experimental procedures were performed according to the method of Abramovitz et al. [23]. [^3^H]PGE_2_ (PerkinElmer, Waltham, MA, USA) binding assays for recombinant EP4 receptors were performed in 10 mM 2-(*N*-morpholino)ethanesulphonic acid-KOH buffer, pH 6, containing 10 mM MgCl_2_ and 1 mM CaCl_2_. Ten micrograms of protein from membrane fractions were incubated in a total volume of 0.1 ml with 1–1.5 nM [^3^H]PGE_2_. To determine the total binding or non-specific binding, 1% dimethyl sulphoxide (DMSO) or 1 μM PGE_2_ was added to the reaction mixture. Incubation was carried out in 96-well multi-well plates for 90 minutes at room temperature prior to separation of the bound and free radioligand by rapid filtration on glass-fibred filters (UniFilter-96 GF/B; PerkinElmer) pre-soaked in 0.3% polyethylenimine. Filters were washed with ice-cold buffer, pH 7.4 (50 mM 4-(2-hydroxyethyl)-1-piperazineethanesulfonic acid [HEPES], NaCl 500 mM, BSA 0.1%), and dried for 30 minutes at 30 °C, then 0.2 ml of MicroScint-20 (PerkinElmer) was added. The residual [^3^H]PGE_2_ binding was determined using a solid scintillation counter (TopCount; PerkinElmer) after at least 1 h of stabilisation. In saturation binding studies, isotherm-binding curves generated with six to eight different concentrations of [^3^H]PGE_2_ (from 0.5 to 25 nM) were evaluated. In competition experiments, curves of compound under investigation ranged from 3 × 10^−10^ M to 10^−6^ M (eight concentration points). In both experimental protocols, all concentration points were performed in duplicate.

### Functional assay in HEK293 cells transfected with EP4 receptor

In studies of CR6086 as a functional receptor antagonist, we took advantage of the evidence that activated EP4 receptors couple with G_s_ proteins to stimulate adenylyl cyclase and increase cyclic adenosine monophosphate (cAMP) cellular levels. Cell membranes prepared from the HEK293 cell line were suspended in 1 ml of stimulation buffer (Hanks’ balanced salt solution 1× + BSA 0.1% + 3-isobutyl-1-methylxanthine 0.5 mM + HEPES 5 mM + MgCl_2_ 10 mM + guanosine triphosphate 1 nM + guanosine diphosphate 10 μM + adenosine triphosphate 100 μM, pH 7.4). The AlphaScreen cAMP functional assay (PerkinElmer) was used according to the manufacturer’s instructions. Because AlphaScreen beads are light-sensitive, the cAMP assay was performed under subdued laboratory lighting, and any incubation involving the beads was carried out in the dark. The assay is based on the competition between endogenous cAMP and exogenously added biotinylated cAMP. The capture of cAMP is achieved by using a specific antibody conjugated to donor beads. Cell membranes were dispensed into white 384-well microplates at a final concentration of 1 μg per well. After 30-minute incubation at room temperature (22–23 °C) in the dark, the biotinylated cAMP and donor beads were dispensed into each well to start the competition reaction. After 1 h of incubation, the plate was read using the EnVision platform (PerkinElmer) to determine the cAMP level (excitation 680 nm, emission 520 or 620 nm). Antagonism was studied by stimulating HEK293 cell membranes with 3 nM PGE_2_. CR6086 was dissolved in 100% DMSO to a final maximal concentration of 0.01% DMSO. A cAMP standard curve was always present in each experiment (concentration range from 1 × 10^−6^ to 1 × 10^−11^ M) with a positive control (no cAMP). Each dilution was prepared in triplicate: 5 μl of anti-cAMP acceptor beads solution, 5 μl of cAMP dilution and 15 μl of biotinylated cAMP/streptavidin donor bead detection mix.

### In vitro evaluation of immunomodulatory and anti-angiogenic potential

#### Gene expression analysis of inflammatory mediators in THP-1 cells differentiated to macrophages

The human monocyte cell line THP-1 was obtained from the ATCC and was grown according to the instructions provided. THP-1 monocytic cells were differentiated to macrophages with 100 nM phorbol 12-myristate 13-acetate (PMA) for 4 days. Macrophages were then stimulated with lipopolysaccharide (LPS) 10 ng/ml and PGE_2_ 10 nM for 3 or 24 h. Total RNA was purified using the ABI Prism 6100 Nucleic Acid PrepStation (Applied Biosystems, Foster City, CA, USA) and retrotranscribed using the High-Capacity cDNA Reverse Transcription Kit (Thermo Fisher Scientific). RT-PCR analysis was performed using the Applied Biosystems 7500 Fast Real-Time PCR System using specific TaqMan assays (number Hs00174131_m1 for IL-6, number Hs00900054_m1 for vascular endothelial growth factor A [VEGFA]; Thermo Fisher Scientific) and, as an endogenous control, the 18S Pre-Developed TaqMan® Assay (Thermo Fisher Scientific). The data analysis, with normalisation on 18S amplified values, was done following Thermo Fisher Scientific’s specific instructions for gene expression relative quantification. All individual data are the result of at least three different analyses for each sample.

#### Source of human peripheral blood mononuclear cells

Whole blood obtained from healthy volunteers upon receipt of their informed consent was used to prepare peripheral blood mononuclear cells (PBMCs). Because the study presented no material ethical issues (samples anonymised and discarded immediately after analysis), the procedure was approved by the internal research ethics advisor.

#### IL-23 release from human dendritic cells

PBMCs were purified using Histopaque (Sigma-Aldrich, St. Louis, MO, USA). CD14^+^ cells were positively selected in a magnetic field (magnetic-activated cell sorting [MACS]; Miltenyi Biotec, Bergisch Gladbach, Germany). Purified CD14^+^ cells were subsequently cultured for 7 days in differentiation medium (RPMI 1640 + 10% FBS, granulocyte-macrophage colony-stimulating factor 50 ng/ml and IL-4 100 ng/ml) and then stimulated with LPS (10 ng/ml), R848 2.5 μg/ml and PGE_2_ 10 nM for 24 h in the presence of CR6086 (0.01–30 μM) or naproxen (1–30 μM). IL-23 release was measured by enzyme-linked immunosorbent assay (ELISA) (R&D Systems, Minneapolis, MN, USA).

#### IL-17 release from human Th17 cells

PBMCs were purified using Histopaque. CD4^+^CD45^+^ naive T cells were indirectly selected in a magnetic field (MACS; Miltenyi Biotec). Purified CD4^+^ cells were subsequently cultured for 7 days in differentiation medium (RPMI 1640 + FBS 10%, IL-6 30 ng/ml, transforming growth factor [TGF]-β 2.25 ng/ml, IL-23 30 ng/ml, IL-1β 20 ng/ml, anti-interferon (IFN)-γ 1 ng/ml and anti-IL-4 2.5 ng/ml). Th17 cell expansion was stimulated by addition of anti-CD3/anti-CD8/anti-CD28 beads to the medium with a cell-to-beads ratio of 1:2. Th17 cells were stimulated with IL-6 30 ng/ml, TGF-β 2.25 ng/ml, IL-23 30 ng/ml, IL-1β 20 ng/ml and PGE_2_ 10 nM for 48 h in the presence of CR6086 (0.03–10 μM) or naproxen (1–10 μM). IL-17 release was measured by ELISA (Abcam, Cambridge, UK).

#### Cell viability

Viability was evaluated by Trypan Blue vital dye count, ViaCount assay (EMD Millipore, Billerica, MA, USA) or MTT [3-(4,5-dimethylthiazol-2-yl)-2,5-diphenyltetrazolium bromide] assay after 24- and 48-h exposure to the test drug.

### Animals, drugs and treatments

Male Lewis rats (Charles River Laboratories, Wilmington, MA, USA) aged 7 weeks and male DBA/1 mice (Charles River Laboratories) aged 6–7 weeks were housed with access to food and water ad libitum in a temperature-controlled room with a 12-h/12-h light/dark cycle at least 1 week before the beginning of the experiment. Experimental procedures were carried out in compliance with national and international laws and policies (Italian Legislative Decree 116/1992, and 26/2014 that transposed the European Union Directive 86/609, and 2010/63/EU on the protection of animals used for scientific purposes) and as authorised by the Italian Ministry of Health (Decr. Min. number 60/2009-B, Decr. Min. number 214/2013-B, and Decr. Min. number 561/2017-PR).

CR6086 (synthesised at Rottapharm Biotech, Monza, Italy), rofecoxib (Sigma-Aldrich) and naproxen (Sigma-Aldrich) were dissolved in distilled water. Tofacitinib (Sigma-Aldrich) was suspended in hydroxypropyl methylcellulose 0.5% Tween 80/0.1% phosphate buffer (150 mM), pH 7. Etanercept (Enbrel; Amgen, Thousand Oaks, CA, USA) and methotrexate (MTX) (Sigma-Aldrich) were dissolved in sterile PBS and sterile saline (NaCl 0.9%), respectively. CR6086, rofecoxib, naproxen and tofacitinib were administered orally; etanercept and MTX were injected intraperitoneally. Comparators were used at doses or dose ranges reaching their maximum pharmacological effects. Volumes of administration were 5 ml/kg and 10 ml/kg for rats and mice, respectively. Naive, sham and control animals always received the appropriate vehicle.

#### Collagen-induced arthritis in rats

Male Lewis rats were immunised by intradermal injection at the base of the tail with an emulsion containing 150 μg of bovine collagen type II (bCII) in complete Freund’s adjuvant (CFA) or received an injection with incomplete Freund’s adjuvant (sham group). Three weeks after the first immunisation, animals were boosted with the same procedure. Three days after booster injection, arthritis had developed in all animals but the sham group. Oedema was assessed via plethysmometer (Ugo Basile, Varese, Italy), and the animals were then randomised to treatment groups. Oedema measurement was performed again after 7 and 14 days of treatment. Animals were blindly scored by two different investigators for clinical signs of arthritis (redness, swelling, pain of hind limb joints) as follows: 0 = normal; 1 = mild but definite redness and swelling of the ankle or wrist or individual digits, regardless of the number of affected digits; 2 = moderate redness and swelling of ankle or wrist; 3 = severe redness and swelling of the entire paw including digits; and 4 = maximally inflamed limb with involvement of multiple joints. The mean of the scores assigned by the investigators was calculated.

Three different experiments in rat CIA are reported: a dose-response study, as well as two comparative studies vs the COX-2 inhibitor rofecoxib and the Janus kinase (JAK) inhibitor tofacitinib, respectively. The number of animals per experimental group is reported in the figure legends.

In the comparative experiment vs rofecoxib, mechanical hyperalgesia was assessed at the end of treatment by using a digital pressure application measurement (PAM) device (Ugo Basile). Increasing pressure was applied to the lateral side of the knee until the animal attempted to escape or vocalised. The force required to elicit this response was measured in grams (gram-force). An optimal stimulus increase rate of 100 g/second was used, and mechanical thresholds were reached after approximately 10 seconds. This increase was visualised with a rate meter on the built-in display, so the pressure applied was visually controlled. At the end of the experiment, animals were killed, and their hind paws were explanted and processed for histology.

#### Histological procedures

Briefly, the paws were fixed in 10% neutral buffered formalin (Bio-Optica, Milan, Italy) and subsequently decalcified in EDTA for 4 weeks. Samples were dehydrated in an ethanol series and embedded in paraffin. At least three non-consecutive sections for each paw were blindly scored at the level of the tarsus, metatarsus and calcaneus for the following histological features: synovial oedema, pannus, synovial inflammatory cell infiltrate, cartilage damage, osteolysis and woven bone. Based on these histological features, a synovial score (synovial oedema, plus synovial inflammatory cell infiltrate, plus pannus), a bone score (woven bone plus osteolysis) and a total score were calculated. Two independent pathologists performed histopathology on blinded slides. The data of right and left hind limbs were summed for each rat.

#### CR6086 pharmacokinetics and pharmacokinetic/pharmacodynamic correlation in rats

CR6086 concentrations in rat plasma were determined by high-performance liquid chromatography (Agilent 1200 series; Agilent Technologies, Santa Clara, CA, USA) coupled with a triple-quadrupole mass spectrometer (API 4000 QTRAP; SCIEX, Concord, ON, Canada). A ZORBAX SB-C18 2.0 × 50-mm, 3.5-μm column (Agilent Technologies) in isocratic conditions was used with mobile phase 0.1% formic acid in water/acetonitrile (68:32 *vol*/vol). The method was linear within the dynamic range 1.0–10,000 ng/ml (lower to upper limit of quantification). CR6086 plasma protein binding was determined in the 0.2–200 μM concentration range using [^14^C]CR6086 and equilibrium dialysis. EP4 receptor occupancy (RO) was calculated on the basis of CR6086 concentrations according to the following formula:

$$ \mathrm{RO}\ \left(\%\right)=\mathrm{Cave}/\left(\mathrm{Cave}+\mathrm{Ki}\right)\times 100, $$

where C_ave_ = AUC_24_/24.

#### Collagen-induced arthritis in mice

Male DBA/1 mice were immunised by intradermal injection at the base of the tail with an emulsion of 200 μg of bCII in CFA containing 3 mg/ml *Mycobacterium tuberculosis* [14, 18, 24, 25]. Non-immunised mice served as the negative control of disease. Animals were monitored by visual inspection for appearance of peripheral oedema. Arthritis onset occurred starting from day 20 after immunisation. Upon onset, animals were recruited and randomised. Recruitment was given a cut-off at day 40. Upon recruitment, arthritis clinical score was assigned, and oedema was measured via caliper. The number of animals per experimental group is reported in the figure legends.

In a first study, mice were randomised into the following treatment groups: vehicle, 30 mg/kg CR6086, 60 mg/kg CR6086, 60 mg/kg naproxen and 10 mg/kg etanercept. Animals received the test drugs for 10 days. CR6086 and naproxen were administered orally once daily, whereas etanercept was administered intraperitoneally every other day. Animals treated with vehicle, 60 mg/kg CR6086, naproxen and etanercept were additionally analysed for the proportion of populations of Th17 cells, Th1 cells, regulatory T cells, B cells, macrophages, neutrophils and dendritic cells by fluorescence-activated cell sorting (FACS) after collection of blood, draining lymph nodes and joints.

In a second study, mice were randomised into the following groups: vehicle, 30 mg/kg CR6086, 60 mg/kg CR6086 and 60 mg/kg naproxen. Animals received the test drugs once daily for 10 days. At the end of the study, serum was isolated for determination of different cytokine biomarkers (IL-6, tumour necrosis factor [TNF]-α, IL-10, IL-17, IFN-γ, IL-22 and IL-23) by multiplex analysis on the MSD platform (Artialis, Liège, Belgium).

In a third study, mice were randomised into the following treatment groups: naive, vehicle, 30 mg/kg CR6086, 1 or 3 mg/kg MTX, with the latter administered alone or in combination with 30 mg/kg CR6086. CR6086 was administered orally once daily. MTX was administered intraperitoneally three times per week (every third day). Mice were treated with test drugs for 16 days.

Oedema measurement was performed every day before treatment, and all animals were blindly scored for clinical signs of arthritis as follows: 0 = normal; 1 = slight swelling and/or erythema; 2 = pronounced oedematous swelling; and 3 = ankyloses and severe swelling. A score of 0.5 was given to swollen toe/toes or when inflammation was localised to one part of the foot. Each limb was measured separately, with a final score based on the sum of the scores from all four paws.

Because animals were recruited for treatment at disease onset, arthritis was already evident in terms of both oedema and clinical score. Therefore, the individual progress of signs was calculated for each animal as the AUC from randomisation (baseline) to the end of treatment.

At the end of the treatment period, animals were killed, and their paws were explanted and processed for histology. In the third study, the serum concentrations of immunoglobulin G (IgG) antibodies against bCII were measured by ELISA (catalogue number 2032; Chondrex, Redmond, WA, USA).

#### Histological procedures

In the second experiment, the signal limb (i.e., the limb that determined the onset of arthritis) was assessed. In the third experiment, instead, all four limbs were analysed, and a summed score for all limbs was calculated. Paws were processed according to the procedure described for rats. At least two non-consecutive sections for each paw, 4 μm thick, of the tarsus, metatarsus, calcaneus, carpal, metacarpal, ulna and radius were collected and assessed for cartilage degeneration, woven bone, osteolysis, synovial inflammation, synovial hyperplasia and pannus formation. Based on these histological features, a synovial score (synovial inflammation, plus synovial hyperplasia, plus pannus), a bone score (woven bone plus osteolysis) and a total score were calculated. Two independent pathologists performed histopathology on blinded slides.

### Statistical analysis

#### In vitro assays and models

Specific binding to the receptors was defined as the difference between total binding and non-specific binding, as determined in the presence of an excess of unlabelled ligand. Binding isotherm curves were analysed to determine the equilibrium binding parameters, affinity (dissociation constant [*K*_d_]) and maximal binding capacity of labelled ligand. Competition curves were analysed to assess the half-maximal inhibitory concentration (IC_50_). The affinity of test compounds (inhibition constant [*K*_i_]) was then calculated according to the Cheng-Prusoff equation: *K*_i_ = IC_50_/(1 + [C]/*K*_d_). Data were analysed by non-linear curve fitting using Prism for Windows version 6 software (GraphPad Software, La Jolla, CA, USA). The results of replicated curves were expressed as mean ± SEM.

#### In vivo models

Statistical analysis using Prism for Windows version 6 software was performed with Student’s *t* test to compare vehicle vs sham and with analysis of variance (ANOVA) or the Kruskal-Wallis test followed by appropriate multiple comparisons tests vs vehicle-treated animals. Variables measured in sham animals were always statistically different from those measured in vehicle animals in all tests at all time points. Unless otherwise stated, data are reported as mean ± SEM. Histological scores are depicted as box-and-whisker plots. The boundary of the box closest to zero indicates the 25th percentile; a line within the box marks the median; and the boundary of the box farthest from zero indicates the 75th percentile. Whiskers (error bars) above and below the box indicate the 90th and 10th percentiles, respectively. All data were considered for statistical comparison of significance.

## Results

### Binding studies

**Fig. 1:**
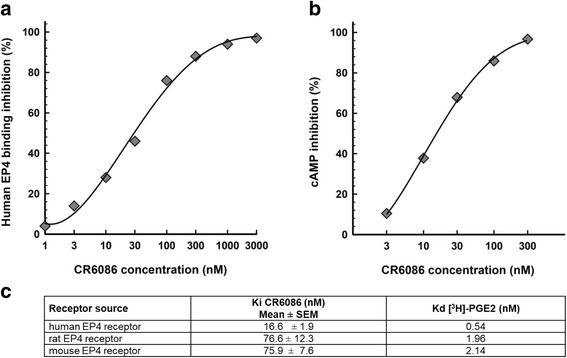
Biochemical characterisation of CR6086 activity on prostaglandin E_2_ receptor 4 (EP4) receptors. **a** Effect of CR6086 on prostaglandin E_2_ (PGE_2_) binding to EP4 receptor. Membranes from human embryonic kidney cells 293 (HEK293) cells transfected with human EP4 receptor were incubated with [^3^H]PGE_2_ and CR6086 (range 1–3000 nM). The results are expressed as percentage inhibition of specific binding. Data are representative of five independent experiments. **b** Effect of CR6086 on cyclic adenosine monophosphate (cAMP) release from membranes of HEK293 cells transfected with human EP4 receptor and stimulated with 3 nM PGE_2_ in the presence of CR6086 (range 3–300 nM). Data are representative of three independent experiments. **c** CR6086 inhibition affinity constant [*K*_i_], and [^3^H]PGE_2_ dissociation constant [*K*_d_] towards human and rodent EP4 receptors

CR6086 stems from a target-based screening program where the affinity of test compounds for the EP4 receptor was determined by radioligand binding assays of membranes from HEK293 cells engineered to express the human, rat or mouse receptor. CR6086 displaced [^3^H]PGE_2_ binding to human EP4 receptors in a concentration-dependent manner, with a *K*_i_ in the low nanomolar range (Fig. 1a). After addition of 0.5% BSA in the assay buffer, the affinity of CR6086 for the human EP4 receptor was similar to that found in the absence of BSA (*K*_i_ values of 15.5 and 16.6 nM, respectively).

Equilibrium binding experiments in the presence of CR6086 (50 nM) showed a significant fourfold decrease in [^3^H]PGE_2_ affinity with respect to that calculated in the absence of CR6086. Conversely, CR6086 only marginally affected (+20%) the maximal binding capacity of [^3^H]PGE_2_, indicating that the compound acts as a competitive antagonist. The affinity of CR6086 for rat and mouse EP4 receptors was still in the nanomolar range but was lower than that calculated for the human receptor (Fig. 1c). Different affinities for the human vs rodent EP4 receptor were also observed for the endogenous agonist PGE_2_, as confirmed by the equilibrium binding parameters of the labelled ligand (Fig. 1c). These results are in keeping with published data [23, 26–28].

CR6086 at concentrations up to 10 μM did not show any relevant binding affinities for the other eicosanoid receptors (i.e., EP1, EP2, EP3, prostaglandin F_2α_, prostaglandin I_2_, thromboxane A_2_ or cysteinyl leukotrienes). At the same concentrations, CR6086 did not inhibit the enzymatic activity of the COX isoforms COX-1 and COX-2 and had no effects on more than 70 pharmacologically relevant targets, including receptors, ion channels and transporters (*see* Additional file 2).

### cAMP assay in HEK293 cells transfected with EP4 receptor

A pharmacodynamic model was set up to measure the ability of test compounds to antagonise PGE_2_-stimulated cAMP production in HEK293 membranes transfected with the human EP4 receptor. CR6086 behaved as a pure EP4 antagonist in these functional assays. The IC_50_ for CR6086 in the presence of PGE_2_ was 22 nM (Fig. 1b).

### Immunomodulatory and anti-angiogenic potential in cell models

**Fig. 2:**
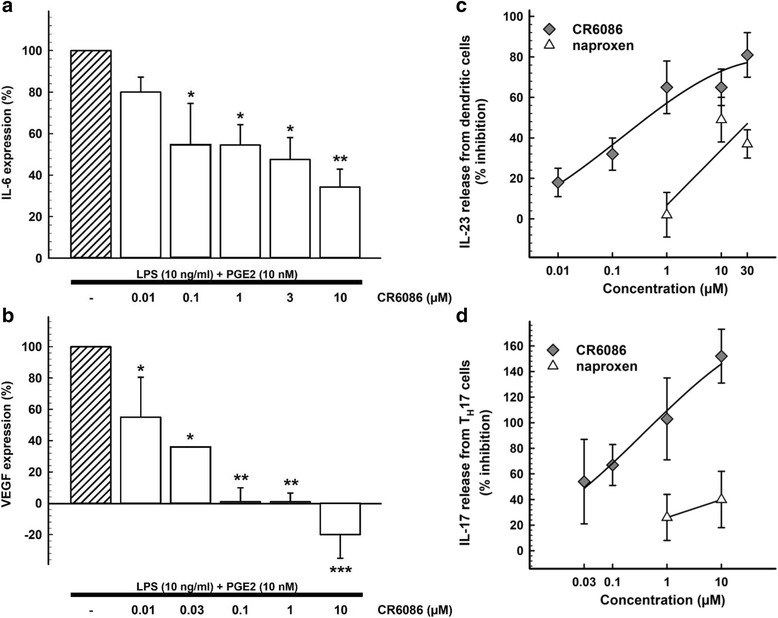
Immunomodulatory and anti-angiogenic effects of CR6086 in vitro. CR6086 inhibition of interleukin (IL)-6 (**a**) and vascular endothelial growth factor (VEGF) (**b**) gene expression in THP-1 cells differentiated to macrophages. CR6086 inhibition of IL-23 release from dendritic cells (**c**) and IL-17 release from Th17 cells (**d**) compared with naproxen. Results are expressed as mean percentage effect/inhibition ± SD of independent experiments performed in triplicate. *P < 0.05 and **P < 0.01 vs control by one-way analysis of variance with Tukey-Kramer multiple comparisons test. *LPS* Lipopolysaccharide, *PGE*_*2*_ Prostaglandin E_2_, *T*_*H*_*17* T-helper type 17 cell

We used different models of immune cell differentiation and expansion to investigate the immunomodulatory, anti-inflammatory and anti-angiogenic properties of CR6086. First, we tested the effects of CR6086 on the gene expression of IL-6 and VEGF in human THP-1 cells differentiated with PMA to macrophages and stimulated with LPS 10 ng/ml + PGE_2_ 10 nM. CR6086 reduced, in a dose-dependent manner, the transcription of IL-6 (Fig. 2a) and VEGF (Fig. 2b). IC_50_ values were 70 nM and 25 nM, respectively. These findings are in agreement with recent evidence on the role of EP4 signalling in promoting IL-6 expression and VEGF-driven angiogenesis [13, 29, 30].

Next, we studied the effects of CR6086 (0.01–30 μM) on PGE_2_-stimulated release of IL-23 from human CD14^+^ cells differentiated to dendritic cells. Naproxen (1–30 μM) served as a control for changes associated with general inhibition of prostaglandin synthesis. CR6086 dose-dependently reduced the release of IL-23 with an IC_50_ of 608 nM, whereas inhibition of IL-23 release by naproxen reached a plateau of about 50% at 10 μM (Fig. 2c).

Finally, we examined whether CR6086 (0.03–10 μM) was able to inhibit PGE_2_-stimulated release of IL-17 from human CD4^+^ T cells differentiated in culture to Th17 cells. CR6086 dose-dependently decreased the release of IL-17 at 48 and 72 h, with 100% inhibition at 1 μM. Naproxen (1–10 μM) was inactive in this assay (Fig. 2d). These data confirm that the EP4 antagonist CR6086 can effectively control the IL-23/IL-17 axis.

### Collagen-induced arthritis in the rat

**Fig. 3:**
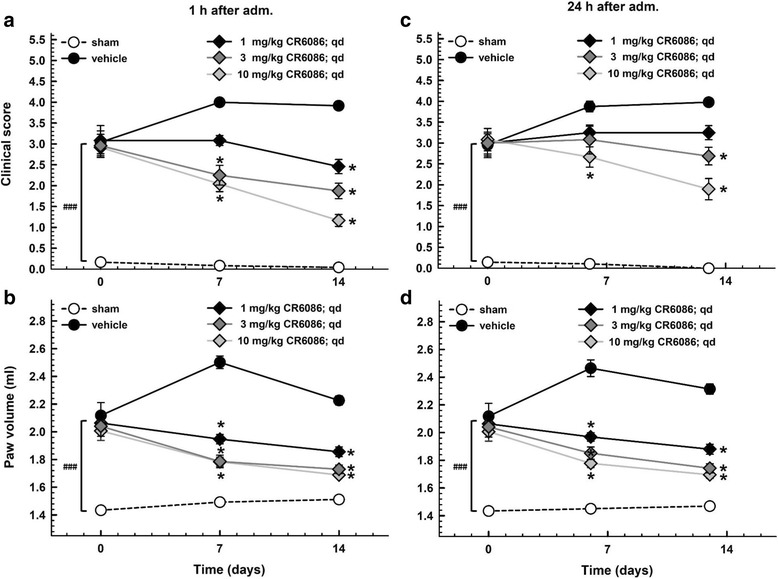
Effect of repeated administration of CR6086 on clinical score and oedema in arthritic rats (collagen-induced arthritis [CIA] model). Arthritis was induced by intradermal injection of type II collagen as described in the Methods section of the main text. Three weeks later, animals were boosted. Daily treatments with oral CR6086 started 3 days after boosting and lasted 14 days. Clinical score (**a** and **c**) and paw volume (**b** and **d**) were assessed 1 h (**a** and **b**) and 24 h (**c** and **d**) after drug administration. Data represent the mean ± SEM of 12 rats/group. *P < 0.05 vs CIA rats treated with vehicle by two-way repeated measures analysis of variance with Tukey’s multiple comparisons test. ^###^*P* < 0.001 CIA vs sham animals at onset (day 0)

**Fig. 4:**
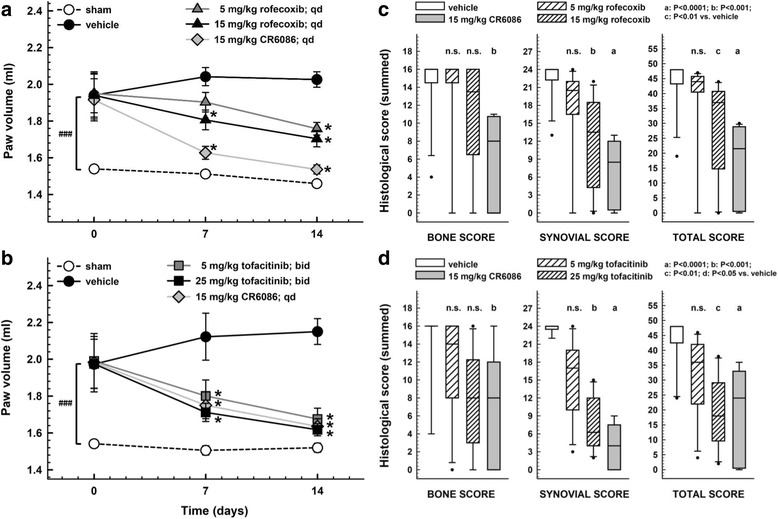
Effect of repeated administration of CR6086, rofecoxib and tofacitinib in arthritic rats (collagen-induced arthritis [CIA] model). Arthritis was induced by intradermal injection of type II collagen as described in the Methods section of the main text. Three weeks later, animals were boosted. Oral treatments were administered daily (CR6086 and rofecoxib) or twice daily (tofacitinib), starting 3 days after boosting, and lasted 14 days. Paw volume (**a** and **b**) was measured 1 h after drug administration. Data represent the mean ± SEM of 12 rats/group. *P < 0.05 vs CIA rats treated with vehicle by two-way repeated-measures analysis of variance with Tukey’s multiple comparisons test. ^###^*P* < 0.001 CIA vs sham animals at onset (day 0). **c** and **d** Box–and-whisker plots with the median histological scores assigned to the hind paws of each rat. ^a^*P* < 0.0001, ^b^*P* < 0.001, ^c^*P* < 0.01, ^d^*P* < 0.05 vs CIA rats treated with vehicle by Kruskal-Wallis test with Dunn’s multiple comparisons test

The CIA model was used to test CR6086 as a DMARD. We investigated the dose response of CR6086 and its efficacy compared with that of rofecoxib, a selective COX-2 inhibitor, and tofacitinib, the first selective JAK inhibitor approved for the treatment of rheumatoid arthritis [31]. At steady state after repeated doses, CR6086 significantly and dose-dependently reduced arthritis clinical score and oedema in CIA rats. Notably, the results were similar at 1 h post-dosing (Fig. 3a and b) and at 24 h post-dosing (i.e., immediately before the next administration) (Fig. 3c and d). Maintenance of the effects throughout the dosing interval indicates that CR6086 acted as a true disease modifier in this model in the dose range from 1 mg/kg (the minimum effective dose) to 10 mg/kg. Increasing the dose to 15 mg/kg, as done in additional experiments including head-to-head studies vs active comparators (Fig. 4), did not further increase the efficacy of CR6086, which plateaued around 10 mg/kg.

CR6086 was more effective than rofecoxib (dose range 5–15 mg/kg once daily) and at least as effective as tofacitinib (dose range 10–50 mg/kg/day given as 5–25 mg/kg twice daily) (Fig. 4). It has been shown that NSAIDs, including both non-selective and selective COX-2 inhibitors, are somewhat effective in the CIA model in rats [32, 33]. In keeping with this, the selective COX-2 inhibitor rofecoxib at 15 mg/kg had a small effect on clinical score and improved to a slight but significant extent the inflammatory parameters (not the structural ones) as evaluated macroscopically, by oedema measurement, and histologically (Fig. 4a and c). The 15 mg/kg dose of rofecoxib also reduced mechanical hyperalgesia in arthritic rats, as assessed with PAM at the end of treatment (Table 1). At variance with rofecoxib, 15 mg/kg CR6086 significantly decreased mechanical hyperalgesia after only 7 days of treatment. At day 14, a complete reversion was attained in arthritic rats on CR6086 (Table 1).

**Table 1:** Mechanical hyperalgesia: effect of repeated administration of CR6086 and rofecoxib in arthritic rats (collagen-induced arthritis model)

	Day 0 (gram-force)	Day 7 (gram-force)	Day 14 (gram-force)
Sham	1174.59 ± 48.41	1040.32 ± 53.38	1074.66 ± 45.60
Vehicle	894.81 ± 69.51^a^	666.79 ± 49.15^b^	600.42 ± 50.23^b^
15 mg/kg CR6086	850.45 ± 59.57^a^	846.33 ± 43.60^b,c^	1023.09 ± 53.97^d^
15 mg/kg rofecoxib	943.70 ± 73.42^a^	673.18 ± 53.76^b^	799.68 ± 61.15^b,c^

Results are reported as mean ± SEM for each group

^a^*P* < 0.05 vs sham rats

^b^*P* < 0.001 vs sham rats

^c^*P* < 0.05 vs vehicle-treated arthritic rats (one-way analysis of variance [ANOVA] followed by multiple comparisons test)

^d^*P* < 0.001 vs vehicle-treated arthritic rats (one-way ANOVA followed by multiple comparisons test)

Moreover, CR6086 markedly and significantly improved all the structural and inflammatory parameters of the disease and was as effective as, but more potent than, tofacitinib (Fig. 4b and d). The response to 15 mg/kg CR6086 (once daily) was greater than that observed with 5 mg/kg tofacitinib (twice daily; i.e., 10 mg/kg/day) and was similar to that observed with 25 mg/kg tofacitinib (twice daily; 50 mg/kg/day).

### CR6086 pharmacokinetic/pharmacodynamic correlation in rats

**Fig. 5:**
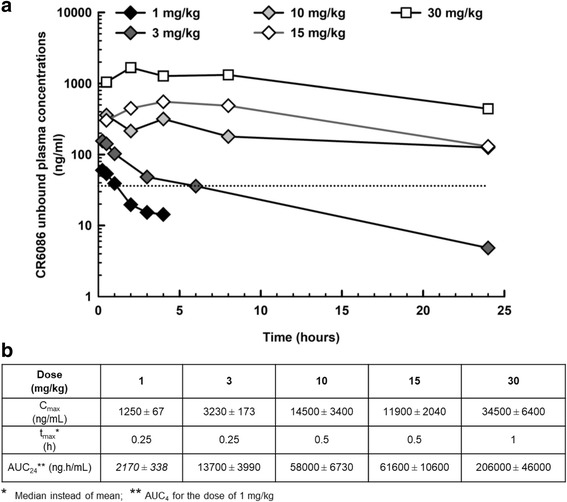
Pharmacokinetics of CR6086 in rats. **a** Unbound concentrations of CR6086 administered orally in the dose range of 1–30 mg/kg. The *dashed line* represents the inhibition constant (36.2 ng/ml) value for the rat prostaglandin E_2_ receptor 4. **b** Pharmacokinetic parameters

The pharmacokinetics of CR6086 were investigated in the animal species used as rheumatoid arthritis models, employing the same administration route and dose range. Preliminary experiments indicated that, in both rats and mice, the pharmacokinetics of CR6086 did not differ between naive and CIA animals. Therefore, for ethical reasons, all pharmacokinetic experiments were conducted in naive animals. In the dose range 1–30 mg/kg in rats, CR6086 was rapidly and completely absorbed following oral administration and was characterised by linear pharmacokinetics (Fig. 5).

Figure 5b reports the unbound (*f*_u_ = 0.0484) CR6086 plasma concentrations determined in rats treated with CR6086 at doses ranging from 1 to 30 mg/kg. The plot also shows the CR6086 *K*_i_ value for the rat EP4 receptor (36.2 ng/ml). At 1 mg/kg, the minimum effective dose in this species (Fig. 3), CR6086 unbound plasma concentrations remained above the *K*_i_ value for up to 1 h post-dosing. The dose of 3 mg/kg, which produced a sustained and clinically relevant disease-modifying effect in the CIA model, was associated with CR6086 unbound plasma concentrations greater than or equal to *K*_i_ for up to approximately 6 h post-dosing. At doses where the efficacy plateaued (i.e., ≥ 10 mg/kg), the unbound CR6086 plasma concentrations remained above the *K*_i_ for the entire dosing interval of 24 h. Because the effects of the 3 mg/kg dose were statistically significant and clinically relevant, an unbound C_ave_ of 27.6 ng/ml over a 24-h dosing interval is a therapeutically relevant exposure in the rat.

An estimation of the receptor occupancy was attempted in the dose range investigated in CIA rats. Because the CIA experiments were conducted after repeated doses, whereas the exposure shown above was determined after a single dose of CR6086, receptor occupancy estimation in the CIA rats took into consideration the accumulation ratio of CR6086 after repeated doses averaging 1.19 in male rats. Based on the CR6086 *K*_i_ of 36.2 ng/ml determined in HEK293 cells expressing the rat EP4 receptor and adjusted for the unbound fraction, the C_ave_ shown above for the dose of 3 mg/kg adjusted for the accumulation ratio upon repeated doses (32.8 ng/ml) is estimated to produce a receptor occupancy approaching 50%. Thus, in our experiments, clinically relevant effects in the CIA rat model were observed starting from approximately 50% EP4 receptor occupancy. As described above, the effects plateaued at doses greater than or equal to 10 mg/kg (Figs. 3 and 4), where indeed the estimated EP4 receptor occupancy ranges from 80% to 93%.

### Collagen-induced arthritis in mice

COX inhibitors are effective in the rat CIA model but not in the mouse (DBA/1) CIA model [34–37]. Thus, the first study in mice exploited this feature to differentiate CR6086 as a DMARD from COX inhibitors as simple anti-inflammatory agents.

**Fig. 6:**
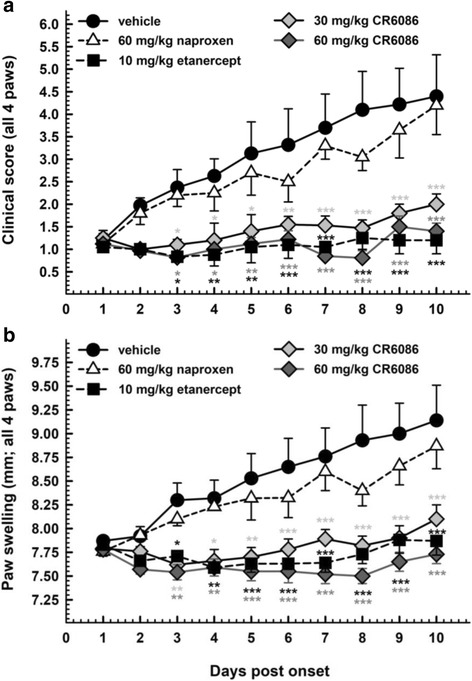
Effect of repeated administration of CR6086, naproxen and etanercept in arthritic mice (collagen-induced arthritis [CIA] model). Arthritis was induced by intradermal injection of bovine type II collagen in complete Freund’s adjuvant as described in the Methods section of the main text. Upon arthritis onset, animals were randomised into experimental groups and treated for 10 days. Data represent clinical score (**a**) and paw oedema (**b**) values assigned daily (mean ± SEM; *n* = 10 mice/group). *P < 0.05, **P < 0.01, ***P < 0.001 vs CIA mice treated with vehicle by two-way analysis of variance with multiple comparisons

Naproxen and etanercept were used as a prototype NSAID and biological DMARD, respectively. Arthritis appeared, on average, after 25 days. Both doses of CR6086, as well as etanercept, significantly improved the disease parameters compared with vehicle-treated CIA mice, as assessed by visual clinical score (Fig. 6a) or paw swelling (Fig. 6b). Naproxen was ineffective in this model.

Consistent with the reported in vitro effects of EP4 antagonism on Th cell subpopulations [11, 14], FACS analysis (Table 2) showed that CR6086 (60 mg/kg) significantly reduced Th1 and Th17 cells in the joint. A parallel decrease in macrophages was also demonstrated. Etanercept (10 mg/kg) decreased only Th1 cells. Naproxen (60 mg/kg) was ineffective.

**Table 2:** Fluorescence-activated cell sorting analysis of cell populations in the joint

Cell population	Percentage of total cells			
	Vehicle	Etanercept	CR6086	Naproxen
Th1	0.32 ± 0.03	0.20 ± 0.05^a^	0.14 ± 0.03^b^	0.35 ± 0.07
Th17	0.20 ± 0.03	0.16 ± 0.08	0.06 ± 0.01^b^	0.21 ± 0.08
Treg	0.54 ± 0.12	0.39 ± 0.10	0.38 ± 0.12	0.63 ± 0.22
B cells	5.00 ± 0.53	5.74 ± 0.68	6.65 ± 0.72	5.77 ± 1.16
Neutrophils	19.04 ± 4.23	10.53 ± 2.81	9.98 ± 2.89^a^	16.47 ± 4.48
Macrophages	20.81 ± 4.44	12.31 ± 5.19	11.14 ± 2.93^a^	16.31 ± 4.44
Dendritic cells	2.31 ± 0.62	1.51 ± 0.18	1.72 ± 0.44	1.96 ± 0.48

*Th* T-helper cell, *Treg* Regulatory T cell

Results are reported as mean percentage ± SEM in each group

^a^*P* < 0.05 vs collagen-induced arthritis (CIA) mice treated with vehicle by paired two-tailed *t* test

^b^*P* < 0.01 vs CIA mice treated with vehicle by paired two-tailed *t* test

**Fig. 7:**
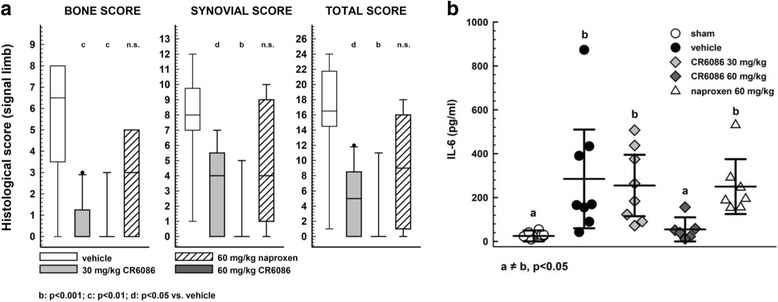
Effect of CR6086 administration compared with naproxen in arthritic mice (collagen-induced arthritis [CIA] model). Arthritis was induced by intradermal injection of bovine type II collagen in complete Freund’s adjuvant as described in the Methods section of the main text. Upon arthritis onset, animals were randomised into experimental groups. Drugs were administered orally once daily for 10 days. **a** Box-and-whisker plots and median of the histological scores for the signal limb of each mouse under the protocol described in the Methods section of the main text. ^b^*P* < 0.001, ^c^*P* < 0.01, ^d^*P* < 0.05 vs vehicle-treated rats by Kruskal-Wallis test followed by Dunn’s test for multiple comparisons. **b** Effect of 30 and 60 mg/kg oral administration of CR6086, and 60 mg/kg naproxen, on interleukin (IL)-6 concentration in sera of arthritic mice at the end of treatment. **a** **≠** **b ***P* < 0.05 by one-way analysis of variance followed by Dunnett’s test

There were similar results in terms of paw swelling and clinical score in a second experiment, which compared the effects of CR6086 and naproxen on histology in the signal limb (i.e., the limb where arthritis developed first). Histological scores confirmed that, unlike NSAIDs, CR6086 acts as a disease modifier in mice (Fig. 7a). In this experiment, we also measured the concentrations of relevant cytokines (i.e., IL-6, TNF-α, IL-10, IL-17, IFN-γ, IL-22 and IL-23) in the serum taken at the time mice were killed. IL-22 and IL-23 were not detectable, perhaps owing to the late collection time point (i.e., more than 5 weeks after immunisation). CR6086 treatment did not affect the serum concentrations of IL-10 in CIA animals, whereas TNF-α, IL-17 and IFN-γ were marginally reduced without reaching statistical significance vs the respective values in vehicle-treated CIA mice (*see* Additional file 3). There was instead a striking effect of the compound on IL-6. The mean serum concentrations of this cytokine, much higher in arthritic animals treated with vehicle than in sham mice, returned towards normal levels in arthritic animals treated with 60 mg/kg CR6086 (Fig. 7b).

The third CIA experiment in this species had the specific objective of investigating the effect of CR6086 and MTX as a combination DMARD therapy. When administered as monotherapy, 30 mg/kg CR6086 significantly reduced arthritis clinical score and oedema within the first week of treatment. MTX at 3 mg/kg modestly reduced the clinical signs over the second week of treatment, whereas 1 mg/kg MTX was inactive (*see* Additional file 4). When combined, CR6086 and MTX strongly and significantly reduced arthritis clinical score and oedema within the first week of treatment, showing clear improvements over MTX alone and over CR6086 alone.

**Fig. 8:**
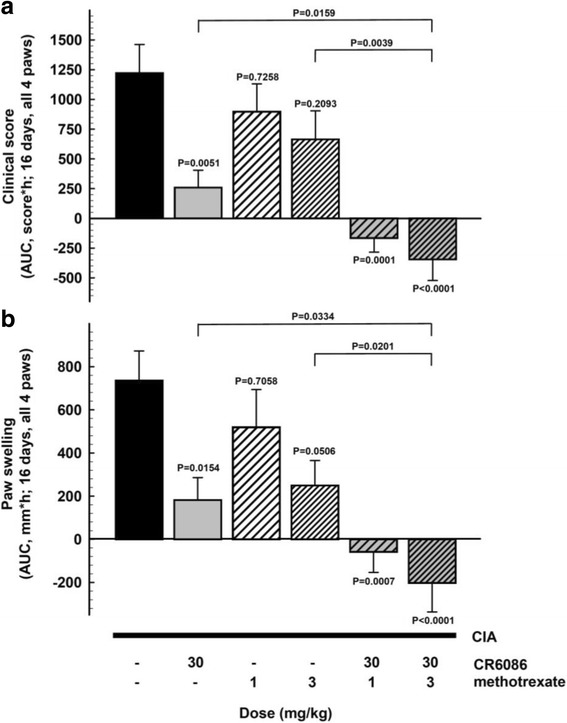
Effect of repeated administration of CR6086, methotrexate (MTX), and their combination in arthritic mice (collagen-induced arthritis [CIA] model). Mice immunised with bovine type II collagen as described in the Methods section of the main text were recruited upon arthritis onset and randomised into experimental groups. Animals were treated with test drugs for 16 days. CR6086 was administered orally once daily, whereas MTX was administered intraperitoneally every third day. Clinical score (**a**) and paw volume (**b**) are expressed as AUC values from randomisation to the end of treatment. Data represent the mean ± SEM of 12 mice/group. *P* values vs vehicle-treated mice are indicated over each bar and were calculated by one-way analysis of variance followed by Dunnett’s test for multiple comparisons. *P* values over connecting lines indicate *t* test comparisons between combination treatment with 3 mg/kg MTX and each single treatment

To better understand how the different treatments had modified the disease course and to normalise for the variability between animals at arthritis onset, we examined disease progression expressed for each mouse as the AUC of clinical signs measured daily from randomisation (baseline) to the end of treatment. AUC analysis of clinical scores (Fig. 8a) and oedema (Fig. 8b) during the whole treatment period showed a clear benefit of CR6086 alone compared with MTX, and especially as an add-on treatment to MTX. The combined treatment indeed not only was effective in slowing arthritis progression but also lowered disease activity below baseline levels, as reflected by negative values in Fig. 8.

**Fig. 9:**
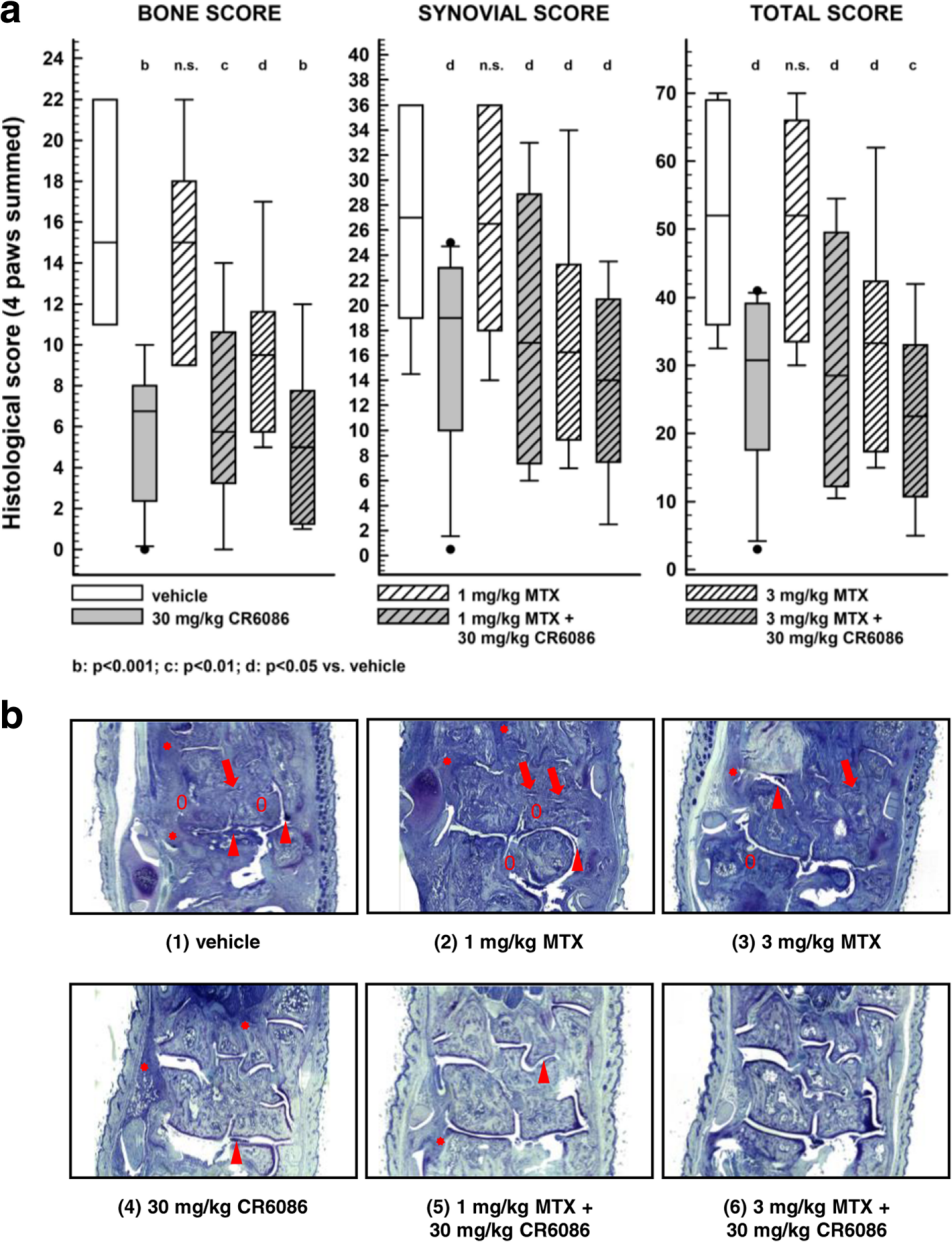
Histological data related to arthritic mice treated with CR6086, methotrexate (MTX), and their combination. Collagen-induced arthritis (CIA) mice, recruited upon arthritis onset, were treated with test drugs for 16 days. CR6086 was administered orally once daily, whereas MTX was administered intraperitoneally every third day. **a** Multiple histological scores of the paws after repeated administration of CR6086, MTX, and their combination. Scores were calculated as summed scores of all the paws under the protocol described in the Methods section of the main text. Data were plotted as box-and-whisker plots. ^b^*P* < 0.001, ^c^*P* < 0.01, ^d^*P* < 0.05 vs vehicle-treated mice by Kruskal-Wallis test followed by two-stage linear step-up procedure of Benjamini, Krieger and Yekutieli. **b** Representative photomicrographs of the paws after repeated administration of CR6086, MTX, and their combination in arthritic mice. Only some representative features are pointed out in the following images. *Arrowhead*: pannus; *arrow*: damaged cartilage; *asterisk*: diffuse inflammation. Elliptic marker (0): osteolysis. (*1*) Hind paw of vehicle control shows all signs of rheumatoid arthritis (RA): cartilage degeneration (*arrows*); pannus formation (*arrowhead*); osteolysis (0) and inflammatory infiltrate (*asterisk*). (*2*) Hind paw from an arthritic animal treated with MTX 1 mg/kg displays all the signs of RA, such as cartilage degeneration (*arrows*), pannus formation (*arrowhead*), osteolysis (0) and inflammatory infiltrate (*asterisk*). Some features, such as woven bone formation and synovial thickening, are not clearly visible at this order of magnification. (*3*) Hind paw from an arthritic animal treated with MTX 3 mg/kg still displays pannus formation (*arrowhead*) and cartilage damage (*arrows*). Moderate inflammation (*asterisk*) and osteolysis (0) can also be observed. (*4*) Hind paw from an arthritic animal treated with CR6086 30 mg/kg. Only few articular joints display minor degeneration (not visible at this magnification); few sites of moderate inflammation (*asterisk*) are present, and moderate pannus formation sites (*arrowhead*) can be seen. (*5*) Hind paw from an arthritic animal treated with CR6086 30 mg/kg + MTX 1 mg/kg displays only minor cartilage degeneration. Very sporadic cases of pannus formation (*arrowhead*) can be observed. (*6*) Hind paw from an arthritic animal treated with CR6086 30 mg/kg + MTX 3 mg/kg shows almost no visible RA features at this magnification

Data from joint histopathology were in line with those derived from clinical signs (Fig. 9). CIA mice showed significant loss of articular cartilage and bone, synovial inflammation and hyperplasia. Treatment with 30 mg/kg CR6086 or 3 mg/kg MTX significantly improved all histological features of arthritis. The combination of CR6086 and 3 mg/kg MTX led to a highly significant improvement in the total combined histological score.

We traced the immunological response of mice by measuring the concentrations of anti-bCII IgG antibodies in their serum at the end of treatment. Anti-bCII IgG antibodies, which are essential for arthritis development in the CIA model [38], were undetectable in sham animals and rose to average levels near 800 μg/ml in the CIA group treated with vehicle (Table 3). As expected, MTX dose dependently reduced anti-bCII IgGs in CIA animals, reaching a statistically significant difference at 3 mg/kg vs vehicle-treated mice. CR6086 at 30 mg/kg was non-significantly less effective than MTX 3 mg/kg. There was only marginal synergism between MTX and CR6086 on this parameter. Their combination did not lead to a further decrease in anti-bCII IgG antibodies, indicating a plateau effect around 200 μg/ml under immunosuppressant treatment.

**Table 3:** Serum levels of collagen II antibodies after 2 weeks of treatment in collagen-induced arthritis mice

Treatments	Anti-collagen II (μg/ml)	*P* value
Vehicle	757.45 ± 89.18	–
30 mg/kg CR6086	324.91 ± 79.82	0.0042
1 mg/kg MTX	483.83 ± 102.82	0.2889
3 mg/kg MTX	242.46 ± 79.35	0.0012
1 mg/kg MTX + 30 mg/kg CR6086	389.50 ± 65.84	0.0314
3 mg/kg MTX + 30 mg/kg CR6086	236.60 ± 50.75	0.0015

*MTX* Methotrexate

*P* values calculated by Dunnett’s multiple comparisons test vs vehicle-treated animals

## Discussion

The present study demonstrates that CR6086 is a novel and selective EP4 receptor antagonist, has favourable immunomodulatory and direct anti-inflammatory properties, and is a highly effective DMARD in rodent models of rheumatoid arthritis. There is now a large body of evidence in the literature about the role of EP4 receptors in the altered immune response observed in experimental autoimmune diseases. Via its EP4 receptor subtype, PGE_2_ functions as a cytokine amplifier (e.g., by enhancing IL-6 expression in macrophages), exerts immunostimulatory effects (e.g., by facilitating Th1 differentiation and Th17 expansion) and promotes angiogenesis (e.g., by inducing VEGF expression in synovial fibroblasts) [11, 13, 14, 39].

Before all of this evidence was available, blockade of EP4 receptors with selective antagonists such as Pfizer’s CJ-042,794 was viewed as a therapeutic strategy merely for the symptomatic relief of pain and inflammation [28]. It was common belief that this new pharmacological class could be safer than conventional NSAIDs, thus representing an alternative to the selective COX-2 inhibitors withdrawn from the market. In addition, there was a tendency to group all drugs interacting with the prostaglandin system as if they were similar to COX-inhibiting NSAIDs, perhaps oversimplifying the pleiotropic effects of prostaglandins. This is particularly true for PGE_2_, which acts on four distinct receptors (EP1–EP4) whose activation can produce superimposing or contrasting effects [40].

More recently, experimental data in cellular and animal models have outlined a far more interesting pharmacological profile for selective EP4 receptor antagonists [11, 41]. Whether they may offer an advantage as simple analgesics over existing drugs such as COX inhibitors is still unclear. Instead, it is now clear that the immunomodulatory effects associated with the use of selective EP4 antagonists in experimental studies cannot be achieved by the general inhibitors of prostaglandin synthesis (i.e., NSAIDs, either non-selective or selective COX-2 inhibitors), nor can they be achieved with selective antagonists for other prostanoid receptors, as also reflected by results from studies of arthritis models in EP4- vs EP(1–3)-knockout mice [18].

As outlined in a recent seminal review [13] and confirmed in our studies of CR6086, EP4 blockade could reduce the structural changes induced by autoimmune diseases at the level of the joint as a whole. Besides its immunostimulatory and pro-angiogenic effects, EP4 is the predominant PGE_2_ receptor in osteoblasts and is expressed in other cells of the joint, including chondrocytes and synovial fibroblasts. PGE_2_ is known to promote both bone resorption and bone formation [42]. However, in rheumatoid arthritis, there is a rapid and sustained osteoclast hyperactivity that supports an early and dramatic bone erosion [10]. EP4 is a major player in this response because it enhances osteoclast formation and activation through RANKL (receptor activator of nuclear factor κB ligand) derived from osteoblasts and from fibroblast-like synoviocytes [10]. Therefore, EP4 antagonists could hinder both the violent bone resorption and the disorganised bony spur formation that characterise some musculoskeletal autoimmune disorders. Altogether, these findings strongly suggest that EP4 receptors play a key role in the pathogenesis of rheumatoid arthritis and are a rational target for the development of novel DMARDs acting on key biochemical pathways involved in the onset and progression of the disease.

We conducted a wide range of in vitro and in vivo studies to characterise the pharmacological profile of the novel EP4 receptor antagonist CR6086. In vitro the compound inhibited the specific binding to EP4 receptors in the nanomolar range in all tested species, with higher affinity to the human recombinant EP4 receptor (*K*_i_ = 16.6 nM). Results obtained from mouse and rat receptor binding assays indicated only a slightly lower affinity, confirming the validity of rodent species in pharmacologic development. Functional assays in cell lines showed that CR6086 is a pure antagonist.

In models of human immune cells in culture, selected for their relevance to the pathogenesis of rheumatoid arthritis, CR6086 markedly decreased PGE_2_-mediated IL-23 release from human dendritic cells, IL-17 release from human Th17 cells, and IL-6 and VEGF transcription in human macrophages. These results showed that EP4 antagonists are well poised to function at multiple cellular levels and at the crossroads of autoimmunity, inflammation and angiogenesis. To our knowledge, the effect of CR6086 on VEGF is not shared by any other synthetic DMARD. It is particularly relevant because VEGF is the most potent pro-angiogenic factor in rheumatoid arthritis [43, 44] and therefore the major driver of synovial hyperplasia and pannus formation [45].

To test the potential of CR6086 as a DMARD, we chose the gold standard paradigm for non-clinical interventional studies in this research area [25]: the CIA model of rheumatoid arthritis in rodents. Oral CR6086 was effective in both rats and mice as well as in all the parameters examined, including oedema, clinical arthritis score and histology. The minimum effective dose in rats was 1 mg/kg, and there was a plateau of maximum efficacy starting at 10 mg/kg, in line with the corresponding receptor occupancy. In these studies, CR6086 was much more effective than NSAIDs and at least as effective as the recently approved JAK inhibitor tofacitinib. In addition, CR6086 showed anti-inflammatory properties targeted to the treatment of immune-mediated inflammatory diseases, as well as promising effects against the immune-mediated disruption of physiologic bone remodelling.

Remarkably, the CIA model in rats is known to respond to NSAID treatment, as confirmed by the results obtained with rofecoxib in the present study, where its efficacy was much lower than that of CR6086. The CIA model in DBA/1 mice instead is resistant to NSAIDs [34–37]. We exploited the different sensitivity between these species to gain more insight into the immunomodulatory activity of CR6086 as distinct from the general anti-inflammatory effects of prostaglandin synthesis inhibitors. A comparative study in mice confirmed the disease-modifying effects of the selective EP4 antagonist under investigation. CR6086 and the biological DMARD etanercept, but not the widely used NSAID naproxen, markedly improved the clinical and histological signs of arthritis in this experimental paradigm. FACS analysis showed that CR6086 significantly reduced macrophages and T-helper cells in the joint, which supports and strengthens in vitro data in the literature regarding the potential of EP4 receptor antagonists as immunomodulators [11, 14]. The blood levels of IL-6, a potent pro-inflammatory agent that plays a crucial role in the pathogenesis of autoimmune diseases [46], were much higher in arthritic mice treated with vehicle than in sham animals. They dropped down towards normal levels in arthritic mice treated with 60 mg/kg CR6086. This dose is greater than that expected to be effective on the basis of differences between mice and rats in terms of EP4 receptor affinity, exposure and unbound fraction in plasma. These findings confirm the different responses to drug therapy in arthritis models in mice (more resistant) and in rats. Notably, even the JAK inhibitor tofacitinib was about eight times less potent in mouse arthritis models than in rat arthritis models (50 mg/kg vs 6.2 mg/kg, respectively, as reported in the FDA pharmacological review).

An important finding in this study is that CR6086 greatly improved the activity of MTX on all parameters measured in the mouse CIA model, supporting the use of CR6086 as, for example, add-on therapy with MTX in DMARD-naive patients with early rheumatoid arthritis. This result was obtained in spite of the fact that the high MTX dose used (3 mg/kg) almost completely blunted the IgG response against bCII, thus confirming that this dose is fully immunosuppressive in mouse CIA. However, the high efficacy of CR6086 administered with the lower (and ineffective) dose of MTX in CIA mice supports the use of this combination DMARD therapy in patients with an inadequate response to MTX.

The potential for CR6086 to relieve painful symptoms in rheumatoid arthritis was tested in the CIA model by PAM in the knee of rats. Mechanical hyperalgesia, which increases progressively in these animals [47, 48], was prevented by CR6086. These results show that disease modification is a major driver, along with targeted anti-inflammatory activity, of the effects of the compound on symptoms. Supporting evidence for the above hypothesis lies in the effects of repeated administration of CR6086 on oedema assessed as paw swelling. There were no differences between measurements at 1 h and at 24 h post-dosing, which would be unlikely if CR6086 acted as a simple anti-inflammatory agent.

## Conclusions

CR6086, a novel selective EP4 receptor antagonist, has proven to be an effective DMARD in rodent species. Its efficacy is associated with a unique ability to interfere with multiple immunological and non-immunological pathways that have a major role in the onset and progression of rheumatoid arthritis. The data we present support the clinical development of CR6086, both as a stand-alone DMARD and as a combination therapy with MTX. This feature makes it possible the use of CR6086 in early rheumatoid arthritis, especially in patients with an aggressive erosive component. The first phase II proof-of-concept trial of CR6086 is ongoing in DMARD-naive patients with early rheumatoid arthritis (ClinicalTrials.gov identifier NCT03163966).

## Additional files

Additional file 1: Chemical structure of CR6086. (DOCX 41 kb)

Additional file 2: Data tables showing the cross-reactivity data for CR6086: 10 eicosanoid receptors, 2 cyclooxygenase isoforms and 76 receptors/transporters/channels. (DOCX 58 kb)

Additional file 3: Data table showing cytokine serum concentrations (pg/ml) of samples determined from 3-PLEX (MSD) in CIA mice. Arthritis was induced in mice by intradermal injection of bovine type II collagen. Upon onset, animals were recruited and randomised into experimental groups. Oral treatments with drugs were administered daily and lasted 10 days. At the end of the study, sera were isolated for determination of indicated cytokines by multiplex analysis on the MSD platform (Artialis, Liège, Belgium). Data represent mean ± SEM of the number of animals per group: *N* = 8 (sham, vehicle, 30 mg/kg CR6086), 6 (60 mg/kg CR6086) and 7 (60 mg/kg naproxen). (DOCX 40 kb)

Additional file 4: Data table showing the effect of repeated administration of CR6086, MTX and their combination in arthritic mice (CIA model). Arthritic CIA mice, recruited upon arthritis onset, were treated with test drugs for 16 days. CR6086 was administered orally once daily, whereas MTX was administered intraperitoneally every third day. Clinical score (**a**) and paw swelling in millimetres (**b**) were reported as median (IQR) and mean (SD), respectively. (DOCX 45 kb)

## Abbreviations

ANOVA: Analysis of variance
ATCC: American Type Culture Collection
bCII: Bovine collagen type II
BSA: Bovine serum albumin
cAMP: Cyclic adenosine monophosphate
cDNA: Complementary DNA
CFA: Complete Freund’s adjuvant
CIA: Collagen-induced arthritis
COX: Cyclooxygenase
CR6086: (*R*)-4-(1-(6-(4-(trifluoromethyl)benzyl)-6-azaspiro[2.5]octane-5-carboxamido)cyclopropyl) benzoate
DMARD: Disease-modifying anti-rheumatic drug
DMSO: Dimethyl sulphoxide
ELISA: Enzyme-linked immunosorbent assay
EP4 receptor: Prostaglandin E_2_ receptor 4
FACS: Fluorescence-activated cell sorting
HEK293: Human embryonic kidney cells 293
hEP4R: Human EP4 receptor
HEPES: 4-(2-Hydroxyethyl)-1-piperazineethanesulfonic acid
IC_50_: Half-maximal inhibitory concentration
IFN-γ: Interferon-γ
IgG: Immunoglobulin G
IL: Interleukin
JAK: Janus kinase
*K*_d_: Dissociation constant
*K*_i_: Inhibition constant
LPS: Lipopolysaccharide
MACS: Magnetic-activated cell sorting
MTX: Methotrexate
NSAID: Non-steroidal anti-inflammatory drug
PAM: Pressure application measurement
PBMC: Peripheral blood mononuclear cell
PGE_2_: Prostaglandin E_2_
PMA: Phorbol 12-myristate 13-acetate
RA: Rheumatoid arthritis
TGF-β: Transforming growth factor-β
Th: T-helper cell
TNF-α: Tumour necrosis factor-α
Treg: Regulatory T cell
VEGF: Vascular endothelial growth factor
