# Supplementary material for: Pharmacological characterisation of CR6086, a potent prostaglandin E2 receptor 4 antagonist, as a new potential disease-modifying anti-rheumatic drug
Source: Arthritis Res Ther. 2018 Mar 1;20:39. doi: 10.1186/s13075-018-1537-8 (PMC5831858; doi:10.1186/s13075-018-1537-8)
Supplement: Supplementary file 1 — Chemical structure of CR6086. (DOCX 41 kb) [file 13075_2018_1537_MOESM1_ESM.docx]

**ADDITIONAL FILE 1**

**Chemical structure of CR6086**

Sodium *(R)-*4-(1-(6-(4-(trifluoromethyl)benzyl)-6-azaspiro[2.5]octane-5-carboxamido)cyclopropyl)benzoate
