# Supplementary material for: Pharmacological characterisation of CR6086, a potent prostaglandin E2 receptor 4 antagonist, as a new potential disease-modifying anti-rheumatic drug
Source: Arthritis Res Ther. 2018 Mar 1;20:39. doi: 10.1186/s13075-018-1537-8 (PMC5831858; doi:10.1186/s13075-018-1537-8)
Supplement: Supplementary file 2 — Data tables showing the cross-reactivity data for CR6086: 10 eicosanoid receptors, 2 cyclooxygenase isoforms and 76 receptors/transporters/channels. (DOCX 58 kb) [file 13075_2018_1537_MOESM2_ESM.docx]

**ADDITIONAL FILE 2**

**Cross-reactivity of CR6086**

Eicosanoid receptors

| **BINDING ASSAYS** | | | | |
| --- | --- | --- | --- | --- |
| **Receptor** | **Radioligand** | **Labelled ligand** | **Test system** | **CR6086**  **Activity at 10µM** |
| EP_1_ | agonist | [^3^H]PGE_2_ | HEK-293 cells | IN |
| EP_2_ | agonist | [^3^H]PGE_2_ | HEK-293 cells | IN |
| EP_3_ | agonist | [^3^H]PGE_2_ | HEK-293 cells | IN |
| FP | agonist | [^3^H]PGF_2α_ | HEK-293 cells | IN |
| IP (PGI_2_) | agonist | [^3^H]iloprost | HEK-293 cells | IN |
| TP (TXA_2_/PGH_2_) | antagonist | [^3^H]SQ 29548 | HEK-293 cells | IN |
| CysLT1 (Cys-LTs) | agonist | [^3^H]LTD4 | HEK-293 cells | IN |
| CysLT2 (Cys-LTs) | agonist | [^3^H]LTD4 | HEK-293 cells | IN |
| **FUNCTIONAL ASSAYS** | | | | |
| **Receptor** | **Effect** | | **Test system** | **CR6086**  **Activity at 10µM** |
| DP_1_ | agonist | | CHO cells | IN |
| DP_1_ | antagonist | | CHO cells | IN |
| IN = < 20% displacement or inhibition (all tables) | | | | |

Cyclooxygenases (enzymatic activity)

| **Enzyme** | **Test system** | **CR6086**  **Activity at 10µM** |
| --- | --- | --- |
| COX-1 | Sf9 cells | IN |
| COX-2 | Sf9 cells | IN |

Receptor/Transporters/Channels characterization panel (binding assays)

| **Receptor** | **Radioligand** | **Labelled ligand** | **Tissue/Species** | **CR6086**  **Activity at 10µM** |
| --- | --- | --- | --- | --- |
| NON-PEPTIDE RECEPTORS | | | | |
| Adenosine receptors | | | | |
| A_1_ | antagonist | [^3^H]DPCPX | Human recombinant CHO cells | IN |
| A_2A_ | agonist | [^3^H]CGS 21680 | Human recombinant HEK-293 cells | IN |
| A_3_ | agonist | [^125^I]AB-MECA | Human recombinant HEK-293 cells | IN |
| Adrenergic receptors | | | | |
| α_1_  (non-selective) | antagonist | [^3^H]prazosin | Rat cerebral cortex | IN |
| α_2_  (non-selective) | antagonist | [^3^H]RX 821002 | Rat cerebral cortex | IN |
| β_1_ | agonist | [^3^H](-)CGP 12177 | Human recombinant HEK-293 cells | IN |
| β_2_ | agonist | [^3^H](-)CGP 12177 | Human recombinant CHO cells | IN |
| Benzodiazepine receptors | | | | |
| BZD (central) | agonist | [^3^H]flunitrazepam | Rat cerebral cortex | IN |
| BZD (peripheral) | antagonist | [^3^H]PK 11195 | Rat heart | IN |
| Cannabinoid receptor | | | | |
| CB_1_ | agonist | [^3^H]CP 55940 | Human recombinant CHO cells | IN |
| Dopamine receptors | | | | |
| D_1_ | antagonist | [^3^H]SCH 23390 | Human recombinant CHO cells | IN |
| D_2S_ | antagonist | [^3^H]methyl-spiperone | Human recombinant HEK-293 cells | IN |
| D_3_ | antagonist | [^3^H]methyl-spiperone | Human recombinant CHO cells | IN |
| D_4,4_ | antagonist | [^3^H]methyl-spiperone | Human recombinant CHO cells | IN |
| D_5_ | antagonist | [^3^H]SCH 23390 | Human recombinant  GH4 cells | IN |
| Muscarinic acetylcholine receptors | | | | |
| M_1_ | antagonist | [^3^H]pirenzepine | Human recombinant CHO cells | IN |
| M_2_ | antagonist | [^3^H]AF-DX 384 | Human recombinant CHO cells | IN |
| M_3_ | antagonist | [^3^H]4-DAMP | Human recombinant CHO cells | IN |
| M_4_ | antagonist | [^3^H]4-DAMP | Human recombinant CHO cells | IN |
| M_5_ | antagonist | [^3^H]4-DAMP | Human recombinant CHO cells | IN |
| Serotonin receptors | | | | |
| 5-HT_1A_ | agonist | [^3^H]8-OH-DPAT | Human recombinant HEK-293 cells | IN |
| 5-HT_1B_ | antagonist | [^125^I]CYP  (+30µM isoproterenol) | Rat cerebral cortex | IN |
| 5-HT_2A_ | antagonist | [^3^H]ketanserin | Human recombinant HEK-293 cells | IN |
| 5-HT_2B_ | agonist | [^125^I](±)DOI | Human recombinant CHO cells | IN |
| 5-HT_2C_ | antagonist | [^3^H]mesulergine | Human recombinant HEK-293 cells | IN |
| 5-HT_3_ | antagonist | [^3^H]BRL 43694 | Human recombinant CHO cells | IN |
| 5-HT_5a_ | agonist | [^3^H]LSD | Human recombinant HEK-293 cells | IN |
| 5-HT_6_ | agonist | [^3^H]LSD | Human recombinant CHO cells | IN |
| 5-HT_7_ | agonist | [^3^H]LSD | Human recombinant CHO cells | IN |
| Histamine receptors | | | | |
| H_1_ | antagonist | [^3^H]pyrilamine | Human recombinant HEK-293 cells | IN |
| H_2_ | antagonist | [^125^I]APT | Human recombinant CHO cells | IN |
| Purinergic receptors | | | | |
| P2X | agonist | [^3^H]α,β-MeATP | Rat urinary bladder | IN |
| P2Y | agonist | [^35^S]dATPαS | Rat cerebral cortex | IN |
| Other non-peptide receptors | | | | |
| GABA (non-selective) | agonist | [^3^H]GABA | Rat cerebral cortex | IN |
| MT_1_ (ML_1A_) | agonist | [^125^I]2-iodomelatonin | Human recombinant CHO cells | IN |
| PCP | antagonist | [^3^H]TCP | Rat cerebral cortex | IN |
| σ  (non-selective) | agonist | [^3^H]DTG | Rat cerebral cortex | IN |
| PEPTIDE RECEPTORS | | | | |
| Angiotensin receptors | | | | |
| AT_1_ | antagonist | [^125^I][Sar^1^, IIe^8^]-AT-II | Human recombinant HEK-293 cells | IN |
| AT_2_ | agonist | [^125^I]CGP 42112A | Human recombinant HEK-293 cells | IN |
| Endothelin receptors | | | | |
| ET_A_ | agonist | [^125^I]endothelin-1 | Human recombinant CHO cells | IN |
| ET_B_ | agonist | [^125^I]endothelin-1 | Human recombinant CHO cells | IN |
| Galanin receptors | | | | |
| GAL_1_ | agonist | [^125^I]galanin | Human recombinant HEK-293 cells | IN |
| GAL_2_ | agonist | [^125^I]galanin | Human recombinant CHO cells | IN |
| Neurokinin receptors | | | | |
| NK_1_ | agonist | [^125^I]BH-SP | U-373MG cells  (endogenous) | IN |
| NK_2_ | agonist | [^125^I]NKA | Human recombinant CHO cells | IN |
| NK_3_ | antagonist | [^3^H]SR 142801 | Human recombinant CHO cells | IN |
| Neuropeptide Y receptors | | | | |
| Y_1_ | agonist | [^125^I]peptide YY | SK-N-MC cells (endogenous) | IN |
| Y_2_ | agonist | [^125^I]peptide YY | KAN-TS cells | IN |
| Opioid receptors | | | | |
| δ_2_ (DOP) | agonist | [^3^H]DADLE | Human recombinant CHO cells | IN |
| κ (KOP) | agonist | [^3^H]U 69593 | Human recombinant CHO cells | IN |
| µ (MOP) | agonist | [^3^H]DAMGO | Human recombinant HEK-293 cells | IN |
| NOP (ORL1) | agonist | [^3^H]nociceptin | Human recombinant HEK-293 cells | IN |
| Other peptide receptors | | | | |
| BB  (non-selective) | agonist | [^125^I][Tyr^4^]bombesin | Rat cerebral cortex | IN |
| B_2_ | agonist | [^3^H]bradykinin | Human recombinant CHO cells | IN |
| CCR1 | agonist | [^125^I]MIP-1α | Human recombinant HEK-293 cells | IN |
| CGRP | agonist | [^125^I]hCGRPα | Human recombinant CHO cells | IN |
| CXCR2 (IL-8B) | agonist | [^125^I]IL-8 | Human recombinant HEK-293 cells | IN |
| MC_4_ | agonist | [^125^I]NDP-α-MSH | Human recombinant CHO cells | IN |
| NTS_1_ (NT_1_) | agonist | [^125^I]Tyr^3^-neurotensin | Human recombinant CHO cells | IN |
| PAC_1_ (PACAP) | agonist | [^125^I]PACAP_1-27_ | Human recombinant CHO cells | IN |
| PDGF | agonist | [^125^I]PDGF BB | Balb/c 3T3 cells | IN |
| sst  (non-selective) | agonist | [^125^I]Tyr^11^-somatostatin-14 | AtT-20 cells | IN |
| TNF-α | agonist | [^125^I]TNF-α | U-937 cells | IN |
| V_1a_ | agonist | [^3^H]AVP | Human recombinant CHO cells | IN |
| VPAC_1_ (VIP_1_) | agonist | [^125^I]VIP | Human recombinant CHO cells | IN |
| NUCLEAR RECEPTORS | | | | |
| AR | agonist | [^3^H]methyltrienolone | LNCaP cells | IN |
| ER | agonist | [^3^H]estradiol | MCF-7 cells | IN |
| GR | agonist | [^3^H]dexamethasone | IM-9 cells  (cytosol) | IN |
| PPARγ | agonist | [^3^H]rosiglitazone | Human recombinant *E.Coli* | IN |
| ION CHANNELS | | | | |
| Ca2+ channel (L, verapamil site) | antagonist | [^3^H]verapamil  [^3^H]D888 | Rat cerebral cortex | IN up to 100µM |
| K_v_ channel | antagonist | [^125^I]α-dendrotoxin | Rat cerebral cortex | IN |
| SK_Ca_ channel | antagonist | [^125^I]apamin | Rat cerebral cortex | IN |
| Cl^-^ channel  (GABA-gated) | antagonist | [^35^S]TBPS | Rat cerebral cortex | IN |
| AMINE TRANSPORTERS | | | | |
| Dopamine transporter | antagonist | [^3^H]BTCP | Human recombinant CHO cells | IN |
| Norepinephrine transporter | antagonist | [^3^H]nisoxetine | Human recombinant CHO cells | IN |
| 5-HT transporter | antagonist | [^3^H]imipramine | Human recombinant CHO cells | IN |
