# Supplementary material for: Pharmacological characterisation of CR6086, a potent prostaglandin E2 receptor 4 antagonist, as a new potential disease-modifying anti-rheumatic drug
Source: Arthritis Res Ther. 2018 Mar 1;20:39. doi: 10.1186/s13075-018-1537-8 (PMC5831858; doi:10.1186/s13075-018-1537-8)
Supplement: Supplementary file 3 — Data table showing cytokine serum concentrations (pg/ml) of samples determined from 3-PLEX (MSD) in CIA mice. Arthritis was induced in mice by intradermal injection of bovine type II collagen. Upon onset, animals were recruited and randomised into experimental groups. Oral treatments with drugs were administered daily and lasted 10 days. At the end of the study, sera were isolated for determination of indicated cytokines by multiplex analysis on the MSD platform (Artialis, Liège, Belgium). Data represent mean ± SEM of the number of animals per group: N = 8 (sham, vehicle, 30 mg/kg CR6086), 6 (60 mg/kg CR6086) and 7 (60 mg/kg naproxen). (DOCX 40 kb) [file 13075_2018_1537_MOESM3_ESM.docx]

**ADDITIONAL FILE 3**

**CIA in mice - Cytokine serum concentrations (pg/mL) of samples determined from 3-PLEX (MSD).**

|  | **Sham** | **Vehicle** | **CR6086 30 mg/kg** | **CR6086 60 mg/kg** | **Naproxen** |
| --- | --- | --- | --- | --- | --- |
| **IFNγ (pg/mL)** | 0.33 ± 0.02 | 2.77 ± 0.36 | 1.79 ± 0.32 | 3.07 ± 0.55 | 3.48 ± 1.10 |
| **TNFα (pg/mL)** | 15.64 ± 1.05 | 44.97 ± 4.41 | 34.37 ± 2.21 | 33.67 ± 3.58 | 43.01 ± 4.55 |
| **IL-10 (pg/mL)** | 12.17 ± 1.65 | 15.38 ± 1.95 | 16.17 ± 1.95 | 18.16 ± 2.65 | 16.21 ± 2.23 |
| **IL-17 (pg/mL)** | 4.54 ± 0.68 | 2.57 ± 0.45 | 2.36 ± 0.62 | 0.92 ± 0.20 | 2.58 ± 0.43 |

Arthritis was induced in mice by intradermal injection of bovine type II collagen. Upon onset, animals were recruited and randomized into experimental groups. Oral treatments with drugs were administered daily and lasted 10 days. At the end of the study, sera were isolated for determination of indicated cytokines by Multiplex analysis on MSD platform (Artialis). Data represent mean ± SEM of N animals/group: N = 8 (sham, vehicle, 30 mg/kg CR6086); 6 (60 mg/kg CR6086); and 7 (60 mg/kg Naproxen).
