# Supplementary material for: Pharmacological characterisation of CR6086, a potent prostaglandin E2 receptor 4 antagonist, as a new potential disease-modifying anti-rheumatic drug
Source: Arthritis Res Ther. 2018 Mar 1;20:39. doi: 10.1186/s13075-018-1537-8 (PMC5831858; doi:10.1186/s13075-018-1537-8)
Supplement: Supplementary file 4 — Data table showing the effect of repeated administration of CR6086, MTX and their combination in arthritic mice (CIA model). Arthritic CIA mice, recruited upon arthritis onset, were treated with test drugs for 16 days. CR6086 was administered orally once daily, whereas MTX was administered intraperitoneally every third day. Clinical score (a) and paw swelling in millimetres (b) were reported as median (IQR) and mean (SD), respectively. (DOCX 45 kb) [file 13075_2018_1537_MOESM4_ESM.docx]

**ADDITIONAL FILE 4**

**Effect of repeated administration of CR6086, MTX, and their combination in arthritic mice (CIA model).**

**A**

| **CLINICAL**  **SCORE**  **(interquartile range)** | **sham** | **vehicle** | **30 mg/kg CR6086** | **1 mg/kg MTX** | **3 mg/kg MTX** | **1 mg/kg MTX +**  **30 mg/kg CR6086** | **3 mg/kg MTX +**  **30 mg/kg CR6086** |
| --- | --- | --- | --- | --- | --- | --- | --- |
| **DAY 0** | 0.0 (0.0 – 0.0) | 2.0 (2.0 – 3.0) * | 2.0 (2.0 – 2.0) * | 3.0 (2.4 – 3.3) * | 2.0 (2.0 – 2.0) * | 3.0 (2.0 – 3.5) * | 2.0 (2.0 – 4.0) * |
| **DAY 1** | 0.0 (0.0 – 0.0) | 3.0 (2.0 – 3.0) | 2.0 (2.0 – 2.0) | 2.8 (2.0 – 3.0) | 2.3 (2.0 – 3.0) | 2.0 (1.0 – 3.1) | 1.5 (1.0 – 2.4) |
| **DAY 2** | 0.0 (0.0 – 0.0) | 3.0 (2.8 – 3.8) | 2.0 (1.0 – 2.0) | 3.0 (3.0 – 4.0) | 3.0 (2.1 – 3.8) | 1.0 (1.0 – 4.1) | 1.5 (1.0 – 2.5) |
| **DAY 3** | 0.0 (0.0 – 0.0) | 4.0 (3.3 – 4.4) | 2.0 (1.0 – 3.1) | 4.0 (3.0 – 4.8) | 3.0 (2.3 – 6.0) | 1.0 (1.0 – 4.6) | 1.0 (1.0 – 2.5) |
| **DAY 4** | 0.0 (0.0 – 0.0) | 4.0 (3.0 – 5.0) | 2.0 (1.0 – 2.3) | 3.5 (3.0 – 5.5) | 4.0 (2.0 – 5.5) | 1.0 (1.0 – 2.5) | 1.0 (1.0 – 2.5) |
| **DAY 5** | 0.0 (0.0 – 0.0) | 4.0 (3.3 – 4.4) | 2.0 (1.3 – 2.0) | 5.0 (3.4 – 6.3) | 3.5 (2.4 – 4.8) | 1.0 (1.0 – 3.0) | 1.8 (0.8 – 2.1) |
| **DAY 6** | 0.0 (0.0 – 0.0) | 4.0 (3.0 – 5.0) | 2.0 (2.0 – 3.0) | 4.8 (3.3 – 8.1) | 4.0 (2.0 – 5.5) | 3.0 (1.0 – 4.0) | 2.0 (1.0 – 2.5) |
| **DAY 7** | 0.0 (0.0 – 0.0) | 5.0 (3.3 – 8.3) | 2.0 (1.0 – 3.1) | 4.5 (2.8 – 7.5) | 4.5 (2.6 – 6.0) | 3.0 (2.0 – 4.3) | 2.5 (1.0 – 4.0) |
| **DAY 8** | 0.0 (0.0 – 0.0) | 5.5 (5.0 – 7.9) | 2.0 (0.5 – 3.5) | 5.5 (3.8 – 7.6) | 4.3 (2.4 – 6.4) | 3.0 (2.0 – 4.0) | 3.0 (1.9 – 3.3) |
| **DAY 9** | 0.0 (0.0 – 0.0) | 6.0 (4.5 – 7.5) | 2.0 (1.0 – 3.3) | 5.0 (3.3 – 7.0) | 4.5 (3.0 – 5.0) | 3.5 (1.0 – 4.0) | 2.5 (1.8 – 3.3) |
| **DAY 10** | 0.0 (0.0 – 0.0) | 5.5 (5.0 – 7.9) | 2.5 (2.0 – 4.3) | 6.5 (3.0 – 7.3) | 5.0 (2.5 – 5.5) | 2.0 (1.0 – 4.0) | 2.0 (1.0 – 3.0) |
| **DAY 11** | 0.0 (0.0 – 0.0) | 6.5 (4.8 – 7.8) | 4.0 (2.0 – 5.0) | 7.3 (4.4 – 7.5) | 5.0 (2.5 – 6.0) | 3.0 (1.0 – 3.0) | 2.0 (0.0 – 3.0) |
| **DAY 12** | 0.0 (0.0 – 0.0) | 7.5 (6.5 – 9.5) | 4.8 (2.8 – 5.1) | 6.8 (5.3 – 7.5) | 5.5 (2.0 – 7.5) | 2.0 (1.0 – 3.4) | 2.0 (0.8 – 2.5) |
| **DAY 13** | 0.0 (0.0 – 0.0) | 7.5 (5.9 – 9.5) | 4.5 (3.0 – 5.5) | 6.3 (4.5 – 7.0) | 6.0 (2.0 – 7.0) | 2.0 (1.0 – 4.1) | 2.0 (0.0 – 2.0) |
| **DAY 14** | 0.0 (0.0 – 0.0) | 7.0 (6.3 – 9.0) | 3.0 (3.0 – 4.9) | 7.3 (5.0 – 7.6) | 4.5 (2.0 – 6.0) | 2.0 (1.0 – 4.0) | 2.0 (0.0 – 3.0) |
| **DAY 15** | 0.0 (0.0 – 0.0) | 6.5 (5.5 – 8.8) | 4.5 (3.0 – 5.0) | 6.5 (5.0 – 7.5) | 4.5 (3.1 – 7.0) | 2.0 (1.0 – 4.0) | 1.0 (1.0 – 2.0) |
| **DAY 16** | 0.0 (0.0 – 0.0) | 7.0 (5.5 – 8.8) | 2.0 (1.0 – 3.5) | 6.5 (5.5 – 8.3) | 4.5 (2.3 – 5.4) | 2.5 (1.0 – 3.0) | 1.0 (1.0 – 2.0) |

*P<0.001 vs. sham animals at the onset (Day 0; Kruskal Wallis and Dunn’s test)

**B**

| **OEDEMA**  **(standard deviation)** | **sham** | **vehicle** | **30 mg/kg CR6086** | **1 mg/kg MTX** | **3 mg/kg MTX** | **1 mg/kg MTX +**  **30 mg/kg CR6086** | **3 mg/kg MTX +**  **30 mg/kg CR6086** |
| --- | --- | --- | --- | --- | --- | --- | --- |
| **DAY 0** | 6.0 (0.8) | 8.5 (0.9) * | 8.3 (0.8) * | 9.3 (0.7) * | 8.9 (1.2) * | 9.1 (1.0) * | 8.8 (1.3) * |
| **DAY 1** | 6.2 (0.4) | 8.7 (1.0) | 8.5 (1.5) | 9.5 (1.3) | 9.0 (1.1) | 8.8 (1.4) | 8.6 (1.4) |
| **DAY 2** | 5.9 (0.6) | 9.3 (1.3) | 8.1 (1.3) | 9.9 (1.5) | 8.8 (1.2) | 8.6 (1.5) | 8.6 (1.4) |
| **DAY 3** | 6.1 (0.5) | 9.6 (1.5) | 8.2 (1.1) | 10.4 (1.5) | 9.4 (1.5) | 8.8 (1.7) | 8.5 (1.4) |
| **DAY 4** | 5.7 (0.3) | 9.7 (1.4) | 8.5 (1.7) | 9.9 (1.7) | 9.3 (1.7) | 8.3 (1.7) | 8.3 (1.4) |
| **DAY 5** | 6.1 (0.4) | 9.4 (1.4) | 8.2 (1.5) | 10.5 (2.2) | 9.6 (1.5) | 8.5 (1.4) | 8.0 (1.4) |
| **DAY 6** | 6.0 (0.4) | 9.8 (2.4) | 8.2 (1.3) | 11.0 (2.8) | 9.6 (2.0) | 9.3 (1.8) | 8.6 (1.3) |
| **DAY 7** | 6.1 (0.4) | 10.6 (2.0) | 8.7 (1.7) | 11.0 (2.5) | 9.8 (2.0) | 9.2 (1.3) | 8.7 (1.4) |
| **DAY 8** | 5.8 (0.4) | 11.0 (2.2) | 8.6 (2.1) | 10.6 (2.1) | 9.7 (2.0) | 9.4 (1.8) | 8.5 (1.6) |
| **DAY 9** | 6.1 (0.6) | 11.0 (2.3) | 9.0 (2.0) | 10.6 (1.6) | 9.7 (1.6) | 9.4 (1.8) | 8.5 (1.3) |
| **DAY 10** | 6.0 (0.4) | 11.1 (2.2) | 9.2 (1.5) | 10.4 (1.8) | 10.1 (2.1) | 9.2 (1.8) | 8.2 (1.3) |
| **DAY 11** | 6.0 (0.4) | 10.8 (2.1) | 9.4 (1.6) | 11.2 (1.9) | 9.6 (1.7) | 9.2 (1.7) | 8.1 (1.4) |
| **DAY 12** | 5.8 (0.3) | 11.4 (2.5) | 10.1 (1.6) | 11.6 (2.2) | 9.6 (2.1) | 8.9 (1.7) | 8.2 (1.1) |
| **DAY 13** | 6.6 (0.3) | 11.5 (1.7) | 9.8 (1.9) | 11.3 (2.2) | 9.6 (1.9) | 9.3 (2.1) | 7.6 (1.7) |
| **DAY 14** | 6.3 (0.4) | 11.8 (1.7) | 9.3 (1.8) | 11.8 (1.8) | 9.6 (2.0) | 9.0 (2.0) | 8.0 (1.3) |
| **DAY 15** | 6.2 (0.3) | 11.5 (1.6) | 9.5 (2.0) | 11.3 (2.4) | 10.1 (1.9) | 9.0 (2.0) | 8.2 (1.6) |
| **DAY 16** | 6.0 (0.4) | 11.5 (1.5) | 9.3 (1.9) | 11.6 (2.0) | 10.1 (1.7) | 8.8 (1.8) | 8.3 (1.6) |

*P<0.001 vs. sham animals at the onset (Day 0; ANOVA and Dunnett’s test)

Arthritic CIA mice, recruited upon arthritis onset, were treated with test drugs for 16 days. CR6086 was administered orally, once a day while MTX was administered intraperitoneally, every third day. Clinical score (Panel A) and paw swelling in mm (Panel B) were reported as median (interquartile range) and mean (SD), respectively.
